# Content validity of the Geriatric Health Assessment Instrument

**DOI:** 10.1590/S1679-45082016AO3455

**Published:** 2016

**Authors:** Rhaine Borges Santos Pedreira, Saulo Vasconcelos Rocha, Clarice Alves dos Santos, Lélia Renata Carneiro Vasconcelos, Martha Cerqueira Reis

**Affiliations:** 1 Universidade Estadual do Sudoeste da Bahia, Jequié, BA, Brazil.; 2 Universidade Federal do Ceará, Fortaleza, CE, Brazil.

**Keywords:** Validation studies, Geriatric assessment, Aged, Aging, Public health

## Abstract

**Objective:**

Assess the content validity of the Elderly Health Assessment Tool with low education.

**Methods:**

The data collection instrument/questionnaire was prepared and submitted to an expert panel comprising four healthcare professionals experienced in research on epidemiology of aging. The experts were allowed to suggest item inclusion/exclusion and were asked to rate the ability of individual items in questionnaire blocks to encompass target dimensions as “not valid”, “somewhat valid” or “valid”, using an interval scale. Percent agreement and the Content Validity Index were used as measurements of inter-rater agreement; the minimum acceptable inter-rater agreement was set at 80%.

**Results:**

The mean instrument percent agreement rate was 86%, ranging from 63 to 99%, and from 50 to 100% between and within blocks respectively. The Mean Content Validity Index score was 93.47%, ranging from 50 to 100% between individual items.

**Conclusion:**

The instrument showed acceptable psychometric properties for application in geriatric populations with low levels of education. It enabled identifying diseases and assisted in choice of strategies related to health of the elderly.

## RESUMO

**Objetivo:**

Avaliar a validade de conteúdo do Instrumento de Avaliação da Saúde do Idoso com baixa escolaridade.

**Métodos:**

Após a elaboração do instrumento de coleta de dados, o questionário foi submetido à avaliação de um comitê de especialistas, formado por quatro profissionais da área da saúde com experiência em pesquisas da epidemiologia do envelhecimento. Os especialistas puderam sugerir questões a serem incluídas/excluídas do instrumento, e avaliar cada bloco do questionário, observando se as dimensões a serem avaliadas foram abrangidas pelos itens do instrumento, em escala intervalar, como “não válida”, “pouco válida” e “válida”. Como medidas para avaliar o grau de concordância do instrumento, foram utilizados o porcentual de concordância e o Índice de Validade de Conteúdo. Considerou-se uma taxa aceitável de concordância o valor de 80%.

**Resultados:**

O instrumento apresentou taxa média de concordância de 86%, variando, dentre os blocos, de 63 a 99% e, dentre os itens, de 50 a 100%. O Índice de Validade de Conteúdo apresentou escore médio de 93,47% e, dentre os itens do questionário, de 50 a 100%.

**Conclusão:**

O instrumento apresentou qualidades psicométricas aceitáveis para ser utilizado entre a população de idosos com baixa escolaridade, permitindo a identificação dos agravos e auxiliando na escolha de estratégias, no que diz respeito à saúde do idoso.

## INTRODUCTION

The elderly population has seen exponential growth over the last decades, in developed and developing countries alike. In Brazil, *e.g.* 650 thousand people enter this age group each year, and most of them show increased vulnerability to chronic diseases. The promotion of improved quality of life among the elderly involves a myriad of Health-related factors, such as functional capacity retention, autonomy, social interaction and self-satisfaction, which must be taken into account, not only from a healthcare perspective, but also as part of a disease prevention and integral approach to geriatric health initiative.^(1)^

The need to recognize elderly people’s true requirements and needs has fostered research in Geriatric Medicine and Health, often aimed to enhance understanding and application of reliable assessment procedures specifically tailored to the elderly population.^(2)^

Questionnaires are among the most valuable data collection tools employed in research, particularly population-based investigation, and is a low-cost, user-friendly data collection alternative.^(2,3)^ However, such instruments must have psychometric properties (validity and reproducibility) that ensure reliability of selected indicators.^(4)^

Instrument validity, including content, criterion and construct validity, is a key dimension and refers to the extent to which a given instrument actually measures what it claims to be measuring. Content validity investigation, in turn, is subjective in nature and is designed to confirm the ability of the approach proposed to reflect certain behaviors in a given sample, thereby determining whether proper item selection, a key step in the development of a new instrument, has been achieved.^(5-8)^

Good, accurate, geriatric health-specific multidimensional questionnaires are scarce, particularly in developing countries, where the elderly population is often quite peculiar. These particularities tend to stand out in less developed areas, such as the Brazilian Northeast, where access to goods and services (*i.e.*, education, health, sanitation, transportation and leisure, among others) is limited.^(9)^

Therefore, if a more comprehensive and effective understanding of the geriatric patient is to be achieved, the reliability of indicators in health assessment instruments must be confirmed.^(5)^

## OBJECTIVE

Assess the content validity of the Elderly Health Assessment Tool with low education.

## METHODS

This study investigated the validity of the Geriatric Health Assessment Instrument (GHAI). The study was conducted in Jequié, Bahia, in October 2013. The city of Jequié is located 365km southwest of Salvador and it ranks 9^th^ in terms of population, in the State of Bahia, with approximately 161,528 inhabitants^(10)^ and a medium (0.66) municipal Human Development Index (HDI).^(11)^

### Instrument construction

The GHAI is a multidimensional questionnaire comprising seven blocks of questions based on previously validated instruments (Appendix 1),^(12-27)^ and its purpose is to investigate different health-related issues in elderly patients participating in the “*Projeto de monitoramento das condições de saúde de idosos”* [Geriatric health monitoring project]; Research Ethics Committee approval number 613.364; CAAE: 22969013.0.0000.0055.

Block 1 (personal and sociodemographic data) comprises 16 questions addressing sex, age, marital state, schooling level, ethnicity/skin color, religion and monthly income. Block 2 (housing conditions) contains four questions addressing housing conditions (house, apartment, shelter, etc.), home ownership and the availability of running water and electricity.

Block 3 addresses general habits and was based on other questionnaires^(12-18)^ inquiring about eating habits, alcohol and tobacco use and level of physical activity. This block contains 18 questions adapted from the model proposed by Fonseca et al.,^(12)^ as well as the instrument used by Munaro.^(13)^ Questions relating to eating habits in the latter instrument (Munaro)^(13)^ were adapted from Nahas et al.^(14)^ while the item referring to behavior change stages was extracted from the model proposed by Prochaska.^(15)^ Questions in this block ask about the frequency of consumption of certain foods in one week (cured meats, industrialized products, deep-fried foods, butter, non-diet soda, sugar, vegetables and fruits). Things like the daily number of complete meals, daily volume of fluid intake, occurrence of digestive problems leading to decreased food intake or appetite loss over the last 12 months, and self-perceived health were investigated. Questions concerning alcohol and tobacco use also address the duration of such habits.

Block 3 questions relating to the level of physical activity were extracted from the International Physical Activity Questionnaire (IPAQ)^(16)^ and the questionnaires proposed by Reichert^(17)^ and Pitanga et al.^(18)^ The questions ask about the time dedicated to physical activities in a typical week, including work- and household-related activities, transport and leisure. The total sitting time in a typical day and the barriers to the practice of physical activities are also investigated, among other issues.

Block 4 (functional capacity) was based on the Kartz^(19)^ and Lawton et al.^(20)^ scales. Questions ask about difficulties in walking or running, climbing stairs, sitting for long periods of time, bending down, extending the arms, using the telephone, shopping, cooking, taking medications, managing finances, etc.

Questions included in Block 5 (health status) were extracted from the World Health Organization Quality of Life Instrument (WHOQOL).^(21)^ In this block, patients are expected to self-assess their health status compared to the past 12 months and to other people of similar age. Current diseases, medications taken and health coverage status are also addressed.

Block 6 (mental health) questions were extracted from the following questionnaires: Memory Complaint Questionnaire (MAC-Q),^(22)^ Mini Mental State Examination (MMSE),^(23)^ Pfeffer Functional Activities Questionnaire,^(24)^ Brazil Old Age Schedule (BOAS),^(25)^ Self-Report Questionnaire (SRQ-20),^(26)^ and the short version of the Yesavage Geriatric Depression Scale (GDS-15).^(27)^ This block addresses temporal orientation, memory, attention level, calculation and language skills. Questions ask about situations potentially experienced in the last 30 days (headaches, difficulty thinking clearly, loss of appetite, etc.), degree of self-satisfaction and mood, among other things.

Finally, Block 7 (quality of life) comprises 52 questions extracted from the WHOQOL^(21)^ questionnaire and inquires about thoughts, feelings and certain aspects related to elderly people’s quality of life.

### Instrument Evaluation

Once completed, the questionnaire was submitted to an expert panel comprising four healthcare professionals (PhDs and PhD students) with a background in research on epidemiology of aging, and chosen on a convenience basis.

Selected professionals were invited to participate in questionnaire validation and content testing. A letter was then sent with instructions regarding the purpose of analyzing the extent to which questionnaire items reflect proposed concepts, the relevance of target objectives and the significance of geriatric health assessment. An Informed Consent Term was also provided.

Evaluators were requested to suggest question inclusion/exclusion and to rate the ability of individual items in questionnaire blocks to encompass target dimensions. An adapted interval scale Hyrkäs et al.^(28)^ was used for this purpose, as follows: not valid (zero to 3 points), somewhat valid (4 to 7 points) or valid (8 to 10 points).

Calculations employed in instrument analysis were based on scores attributed to individual questionnaire items using the aforementioned interval scale. Therefore, rater perception was quantified regardless of variable categorization (*i.e.*, descriptive/non-descriptive). Percent agreement (% agreement = number of participants that fully agreed about a given item/number of participants x 100) and the Content Validity Index (CVI = number of valid answers/total number of answers) were used as measurements of agreement about GHAI content validity.

The minimum acceptable interrater agreement in this study was set at 80%. Valid answer scores employed in CVI calculation corresponded to the sum of agreement scores entered in items rated 4 to 10, bearing in mind the exclusion of items rated 0 to 3 and review of those rated 4 to 7.^(28,29)^

## RESULTS

The appropriateness of items proposed in questionnaire blocks and their respective relevance to measurement of target attributes (*i.e.*, whether the instrument was comprehensive enough to provide a representative sample of the target behavioral domain) was expressed as inter-rater percent agreement.

**Figure 1:**
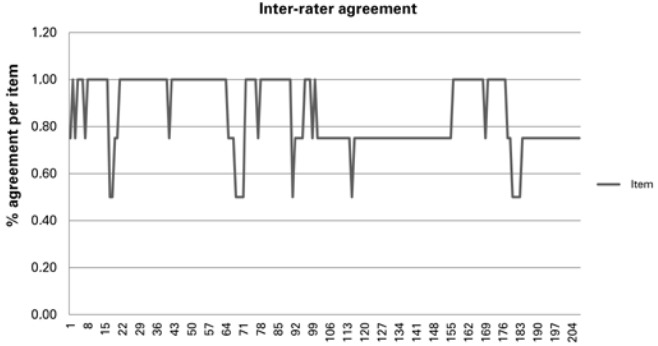
Mean percent agreement score per item

The GHAI in this study achieved a mean percent agreement score of 86%; percent agreement ranged from 63 to 99% (Table 1) and 50 to 100% (Figure 1) between and within blocks respectively.

**Table 1:** Item analysis and percent agreement per instrument block

Blocks	Items	Items excluded	Percent agreement
	(n)	(n)	(%)
1. Personal and sociodemographic data	16	1	95
2. Housing	4	1	63
3. General habits	51	5	94
4. Funcional capacity	19	0	99
5. Health status	11	0	82
6. Mental health	54	39	75
7. WHOQOL-OLD	52	4	83

Total	207	50	86

Inter-rater agreement in his study was therefore considered acceptable (*i.e.*, greater than 80%).

**Figure 2:**
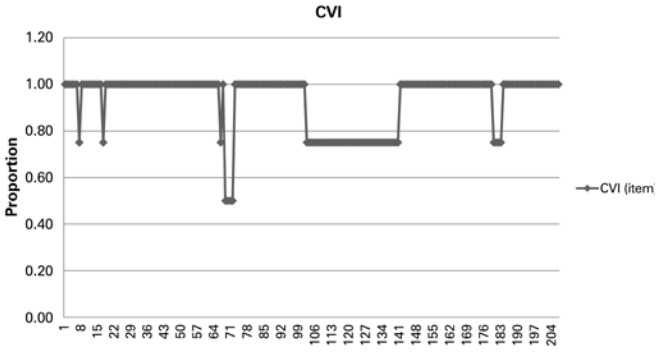
Mean Content Validity Index score per item. Fifty questions were excluded from the questionnaire, as follows: Personal and sociodemographic data (Block 1) - 1 question; Housing (Block 2) – 1 question; General habits (Block 3) – 5 questions; Mental health (Block 6) – 39 questions; WHOQOL-OLD (Block 7) – 4 questions (sections 1, 2, 3, 6 and 7 respectively)

The sum of items rated as relevant yielded a mean CVI score of 93.47, with individual item CVI scores ranging from 50 to 100% (Figure 2). Items rated as non-relevant or non-representative (CVI score <100%) were not considered.

CVI: Content Validity Index.

## DISCUSSION

The major issue facing health care services today is the growing elderly population, whose morbidity profile is dominated by chronic diseases that often lead to functional limitations, with negative impacts on patient’s quality of life.^(19,30)^

This study evaluated a multidimensional geriatric health assessment questionnaire GHAI addressing different geriatric health-related aspects. Satisfactory levels of validity were achieved. Geriatric health assessment instruments are thought to reflect geriatric health status and assist health service managers in making decisions about the best care to be delivered to the population.

Percent agreement in this study was greater than the minimum acceptable value of 80%. The 80% cutoff point was adopted due to the reduced number of evaluators involved, bearing in mind the minimum of five evaluators required to reach a 90% global agreement score.^(4,31)^

Critical analysis led to questionnaire improvement. Some questions were excluded to better adapt the instrument to the target population.

Instrument length (207 Items) was thought to be excessive; therefore, 50 questions rated as non-relevant or non-representative were excluded to facilitate instrument validation and interviewee understanding. Shorter questionnaires are vital for expanded use in public health settings, if contributions to improved geriatric health understanding and care are to be expected.

A study investigating psychometric qualities of psychological and social well-being indicators applied to an elderly population living in Chicago (Chang et al.)^(32)^ suggested shorter, straightforward questionnaires are better suited for population-based health surveys involving elderly people, particularly in low schooling level settings, as in this study.

Some limitations of this study, particularly the reduced number of evaluators, precluded a more robust analysis of the instrument proposed and prevented comparisons with parameters employed in previous trials.

Content validation is a necessary step in novel assessment instrument constructions. However, other psychometric strategies must be considered to overcome the limitations introduced by the subjective nature of expert panel-based approaches.^(4,6,8,33)^ Yet, despite shared experiences, potential individual differences may interfere with instrument sensitivity and ability to properly reflect the health status of all individuals in the target population.

## CONCLUSION

The results of this study suggest that the proposed Geriatric Health Assessment Instrument contains acceptable psychometric indicators of content validity. Therefore, the instrument can be applied in geriatric health surveys targeting populations with low schooling levels, and is a standardized, user-friendly tool for improved geriatric health understanding and appropriate healthcare decision making.

**Appendix 1**. Instrumento de Avaliação de Saúde do Idoso

## References

[1] Veras R (2009). Envelhecimento populacional contemporâneo: demandas, desafios e inovações. Rev Saude Publica.

[2] Virtuoso JS, Santos CA, Ferreira AN, Tribess S (2008). Propriedades psicométricas da Escala de Atividade Física adaptada para mulheres idosas (EAFI). Geriatria & Gerontologia.

[3] Schmitter-Edgecombe M, Parsey C, Lamb R (2014). Development and psychometric properties of the instrumental activities of daily living: compensation scale. Arch Clin Neuropsychol.

[4] Alexandre NM, Coluci MZ (2011). Validade de conteúdo nos processos de construção e adaptação de instrumentos de medidas. Cien Saude Colet.

[5] Rodrigues RM (2008). Validação da versão em português europeu de questionário de avaliação funcional multidimensional de idosos. Rev Panam Salud Publica.

[6] Lacerda TT, Magalhães LC, Rezende MB (2007). Validade de conteúdo de questionários de coordenação motora para pais e professores. Rev Ter Ocup Univ São Paulo.

[7] Ottati F, Noronha AP (2003). Parâmetros psicométricos de instrumentos de interesse profissional. Estudo Pesqui Psicolo.

[8] Rubio DM, Berg-Weger M, Tebb SS, Lee ES, Rauch S (2003). Objectifying content validity: conducting a content validity study in social work research. Soc Work Res.

[9] Morais EP, Rodrigues RA, Gerhardt TE (2008). Os idosos mais velhos no meio rural: realidade de vida e saúde de uma população do interior gaúcho. Texto Contexto Enferm.

[10] Instituto Brasileiro de Geografia e Estatística (IBGE) (2014). Diretoria de Pesquisas. Coordenação de População e Indicadores Socias. Cidades.

[11] Programa das Nações Unidas para o Desenvolvimento. Instituto de Pesquisa Econômica Aplicada, Fundação João Pinheiro (2010). Atlas do Desenvolvimento Humano no Brasil 2013. Ranking - Bahia (2010).

[12] Fonseca MJ, Chor D, Valente JG (1999). Hábitos alimentares entre funcionários de banco estatal: padrão de consumo alimentar. Cad Saude Publica.

[13] Munaro HL (2007). Efetividade de uma intervenção educacional de curta duração sobre a diminuição da prevalência de fatores de risco para doenças e agravos não-transmissíveis.

[14] Nahas MV, Fonseca AS (2004). Estilo de Vida e hábitos de lazer dos trabalhadores da indústria catarinense (1999 – 2004).

[15] Prochaska JO, Marcus BH, Dishman RK (1994). The transtheoretical model: applications to exercise. Advances in exercise adherence.

[16] Matsudo SM, Araújo T, Matsudo VR, Andrade D, Andrade E, Oliveira LC (2001). Questionário Internacional de Atividade Física (IPAQ): estudo de validade e reprodutibilidade no Brasil. Rev Bras Ativ Fis Saude.

[17] Reichert FR (2004). Barreiras à prática de atividades físicas: prevalência e fatores associados.

[18] Pitanga FJ, Lessa I (2005). Prevalência e fatores associados ao sedentarismo no lazer em adultos. Cad Saude Publica.

[19] Duarte YA, Andrade CL, Lebrão ML (2007). O Índex de Katz na avaliação da funcionalidade dos idosos. Rev Esc Enferm USP.

[20] Lawton MP, Brody EM (1969). Assesment of older people: self-maintaining and instrumental activities of daily living. Gerontologist.

[21] Fleck MP, Chachamovich E, Trentini CM (2003). Projeto WHOQOL-OLD: método e resultados de grupos focais no Brasil. Rev Saude Publica.

[22] Crook TH, Feher EP, Larrabee GJ (1992). Assessment of memory complaint in age-associated memory impairment: the MAC-Q. Int Psychogeriatr.

[23] Bertolucci PH, Brucki SM, Campacci SR, Juliano Y (1994). O mini-exame do estado mental em uma população geral. Arq Neuropsiquiatr.

[24] Santos AA, Pavarini SC (2011). Funcionalidade de idosos com alterações cognitivas em diferentes contextos de vulnerabilidade social. Acta Paul Enferm.

[25] Veras R, Dutra S (2008). Perfil do idoso brasileiro: questionário BOAS.

[26] Mari JJ, Williams P (1986). A validity study of a psychiatric screening questionnaire (SRQ-20) in primary care in the city of Sao Paulo. Br J Psychiatry.

[27] Almeida OP, Almeida SA (1999). Confiabilidade da versão brasileira da Escala de Depressão Geriátrica (GDS) versão reduzida. Arq Neuropsiquiatr.

[28] Hyrkäs K, Appelqvist-Schmidlechner K, Oksa L (2003). Validating an instrument for clinical supervision using an expert panel. Int J Nurs Stud.

[29] Topf M (1986). Three estimates of interrater reability for nominal data. Nurs Res.

[30] Pinto RB, Bastos LC (2007). Abordagem das pesquisas em epidemiologia aplicada à gerontologia no Brasil: revisão da literatura em periódicos, entre 1995 e 2005. Rev Bras Epidemiol.

[31] Lynn MR (1986). Determination and quantification of content validity. Nurs Res.

[32] Chang ES, Beck T, Simon MA, Dong X (2014). A psychometric assessment of the psychological and social well-being indicators in the PINE study. J Aging Health.

[33] Sireci SG (1998). The construct of content validity. Soc Indic Res.

