# Supplementary material for: Content validity of the Geriatric Health Assessment Instrument
Source: Einstein (Sao Paulo). 2016 Apr-Jun;14(2):158–77. doi: 10.1590/S1679-45082016AO3455 (PMC4943349; doi:10.1590/S1679-45082016AO3455)
Supplement: Supplementary file 1 [file 1679-4508-eins-14-2-0158_sd.pdf]

Appendix 1. Instrumento de Avaliação de Saúde do Idoso

Instrumento de coleta

Número do questionário |___|___|___|

Nome do entrevistador: ____.

Visita	1	2	3
Data	Dia I___I___I	Dia I___I___I	Dia I___I___I
	Mês I___I___I	Mês I___I___I	Mês I___I___I
	Ano I___I___I___I	Ano I___I___I___I	Ano I___I___I___I

Hora de início: _____:_____

Hora de término: _____:_____

Bloco 1

I. Informações pessoais e sociodemográficas

1. Nome do entrevistado:

2.Sexo:

0 ( ) Feminino

1 ( ) Masculino

3. Endereço completo/telefone:

4. Idade: ___ anos

4.1. Data de nascimento: ___/___/___

5. Situação conjugal atual:

1 ( ) Casado(a) 2 ( ) União estável 3 ( ) Solteiro/a

4 ( ) Divorciado(a)/separado(a)/desquitado(a) 5 ( ) Viúvo/a

6. No total, quantas vezes, o(a) Sr.(a) esteve casado(a) ou em união?

Nº de vezes |___|___|

7. Na escola, qual a última série/grau que concluiu com aprovação?

1 Nunca foi à escola ( )

2 Lê e escreve o nome ( )

3 Fundamental I ( ) 1a ( ) 2a ( ) 3a ( ) 4a

4 Fundamental II ( ) 5a ( ) 6a ( ) 7a ( ) 8a

5 Ensino Médio ( ) 1a ( )2a ( ) 3a

6 Superior ( ) completo ( ) incompleto

8. Como você classificaria a cor da sua pele?

1 ( ) Branca 2 ( ) Amarela (oriental) 3 ( ) Parda

4 ( ) Origem indígena 5 ( ) Preta 9 ( ) NS

9. Cor da pele (entrevistador):

1 ( ) Branca 2 ( ) Amarela (oriental) 3 ( ) Parda

4 ( ) Origem indígena 5 ( ) Preta

10. Quantos filhos e filhas nascidos vivos o(a) Sr.(a) teve? (não inclua enteados, filhos adotivos, abortos ou filhos nascidos mortos).

Número de filhos: |____|____|____(98) NS (99) NR

11. Atualmente o(a) Sr.(a) mora sozinho(a) ou acompanhado(a)?

1 ( ) Acompanhado 2 ( ) Sozinho 3 ( ) NR 4 ( ) NS

12. Desde que o(a) Sr.(a) nasceu viveu no campo por 5 anos ou mais?

0 ( ) Não 1 ( ) Sim 2 ( ) NS 3 ( ) NR

13. Qual é a sua religião?

1 ( ) Católica 2 ( ) Protestante 3 ( ) Judaica

4 ( ) Espírita/kardecista 5 ( ) Umbanda 6 ( ) Outras ___________

14. Qual a importância da religião em sua vida?

(1) Importante (2) Regular (3) Nada importante (8) NS (9) NR

15. Com que frequência o(a) Sr.(a) vai à igreja ou ao serviço religioso?

( ) Nunca ( ) Várias vezes por ano ( ) Uma duas vezes por mês

( ) Quase toda semana ( ) Mais de uma vez por semana ( ) NS ( ) NR

16. Quanto você ganha, em média, por mês? R$ ________ ( ) Não se aplica

continue...

...Continuation

Appendix 1. Instrumento de Avaliação de Saúde do Idoso

**Bloco 2**<br>**II. Características da moradia**	
Agora, quero fazer algumas perguntas sobre a sua casa.	
17. Esta moradia é? (leia as opções até obter uma resposta afirmativa)	
(1) Casa?	(4) Abrigo?
(2) Apartamento?	(5) Outro? Especifique:__________________
(3) Barraco ou *trailer*?	(9) NR
18. Esta casa é: (leia as opções até obter uma resposta afirmativa)	
(1) Própria e quitada, em terreno próprio;	(4) Arrendada ou alugada emprestada;
(2) Ainda está pagando;	(5) Outro? Especifique:____________________
(3) Própria, em terreno que não é próprio;	(8) NS (9) NR
19. A sua casa tem luz elétrica? (1) Sim (2) Não	
20. Os moradores desta casa dispõem de água encanada? (leia as opções até obter uma resposta afirmativa)	
(1) Dentro da casa?	(4) Não dispõem de água encanada?
(2) Fora da casa, mas no terreno?	(3) Fonte pública? (9) NR

Bloco 3

III. Hábitos de vida[12-18]

III.A. Hábitos alimentares

**Alimento**	**4 ou mais vezes por semana**	**1 a 3 vezes por semana**	**Menos de 1 vez por semana**	**Não consome**	**Não sabe informar**
21 - Carnes salgadas: bacalhau, charque, carne seca, carne de sol, paio, toucinho, costela etc.	3	2	1	0	
22 - Produtos industrializados: enlatados, conservas, sucos engarrafados, sucos desidratados, sopa desidratada, produtos em vidro etc.	3	2	1	0	
23 - Embutidos: linguiça, salsicha, fiambre etc.	3	2	1	0	
24 - Frituras	3	2	1	0	
25 - Manteigas	3	2	1	0	
26 - Carne de porco: pernil, carrê, costeleta etc., carne de carneiro ou cabra	3	2	1	0	
27 - Carne de vaca	3	2	1	0	
28 - Refrigerantes não dietéticos	3	2	1	0	
29 - Balas, doces, geleias, bombons e chocolate	3	2	1	0	
30 - Açúcar, mel ou melaço usados como adoçantes no café, chá, suco etc.	3	2	1	0	
31 - Ovos cruz, cozidos, fritos, pochê etc.	3	2	1	0	
32 - Verduras, legumes e frutas	0	1	2	3	

33 - Quantas refeições completas o(a) Sr.(a) faz por dia?				
(1) Uma	(2) Duas	(3) Três ou mais	(8) NS	
34a - Consome leite, queijo ou outros produtos lácteos pelo menos uma vez por dia?				
(1) Sim	(2) Não	(8) NS	(9) NR	
34b - O leite e derivados que você consome são integrais, semidesnatados ou desnatados?				
(1) Integrais	(2) Desnatados	(3) Semidesnatados	(8) NS	(9) NR
35 - Come ovos, feijão ou lentilhas (leguminosas), pelo menos uma vez por semana?				
(1) Sim	(2) Não	(8) NS	(9) NR	
36 - Come carne, peixe ou aves pelo menos três vezes por semana?				
(1) Sim	(2) Não	(8) NS	(9) NR	
37 - Tem comido menos por problemas digestivos ou falta de apetite nos últimos 12 meses?				
(1) Sim	(2) Não	(8) NS	(9) NR	
38 - Quantos copos ou xícaras de líquido consome diariamente? (incluir água, café, leite, chá, suco etc.)				
(1) Menos de 3 copos	(2) De 3 a 5 copos	(3) mais de 5 copos	(8) NS	(9) NR

continue...

...Continuation

Appendix 1. Instrumento de Avaliação de Saúde do Idoso

Bloco 3

III.B. Estágio de mudança de comportamento dos hábitos alimentares

39 - Em quantos dias de uma semana normal o Sr (a) come FRUTAS ou toma SUCO NATURAL DE FRUTAS?

[ ]0 [ ]1 [ ]2 [ ]3 [ ]4 [ ]5 [ ]6 [ ]7

40 - Em quantos dias de uma semana normal o Sr (a) come VERDURAS (saladas verdes, tomate, cenoura, abóbora, etc.)?

[ ]0 [ ]1 [ ]2 [ ]3 [ ]4 [ ]5 [ ]6 [ ]7

41 - Considera-se SAUDÁVEL o consumo diário de frutas e verduras. Em relação a seus hábitos alimentares o Sr (a) considera que:

1[ ] Incluo frutas e verduras diariamente em minha alimentação HÁ MAIS DE 6 MESES

2[ ] Incluo frutas e verduras diariamente em minha alimentação HÁ MENOS DE 6 MESES

3[ ] Não tenho este hábito, mas pretendo incluir frutas e verduras na minha alimentação diária nos próximos 30 DIAS.

4[ ] Não tenho este hábito, mas pretendo incluir frutas e verduras na minha alimentação diária nos próximos 6 MESES.

5[ ] Não tenho este hábito, e não pretendo incluir frutas e verduras na minha alimentação diária nos próximos 6 MESES.

III.C. Consumo de álcool e tabaco

Uso de bebidas alcoólicas

42 - Você consome bebidas alcoólicas? Se você NÃO BEBE, siga para o bloco III D

0 ( ) Sim 1 ( ) Não

43 - Alguma vez sentiu que deveria diminuir a quantidade de bebida ou parar de beber?

0 ( ) Sim 1 ( ) Não

44 - As pessoas o(a) aborrecem porque criticam o seu modo de beber?

0 ( ) Sim 1 ( ) Não

45 - Sente-se chateado consigo mesmo(a) pela maneira como costuma beber?

0 ( ) Sim 1 ( ) Não

46 - Costuma beber pela manhã para diminuir o nervosismo ou a ressaca?

0 ( ) Sim 1 ( ) Não

Hábito de fumar

47 - Você já foi fumante? 0 ( ) Sim 1 ( ) Não

48 - Você fuma atualmente? Se você NÃO FUMA, siga para o bloco III D 0 ( ) Sim 1 ( ) Não

49 - Quantos cigarros você fuma por dia?_____Cigarros

50 - Há quanto tempo você fuma? _____anos_____meses_____dias

III.D. Atividade física

Questionário Internacional de Atividade Física (IPAQ)

As perguntas estão relacionadas ao tempo que você gasta fazendo atividade física em uma semana normal/habitual

Para responder as questões, lembre que:

- Atividades físicas vigorosas são aquelas que precisam de um grande esforço físico e que fazem respirar muito mais forte que o normal.
- Atividades físicas moderadas são aquelas que precisam de algum esforço físico e que fazem respirar um pouco mais forte que o normal.
- Atividades físicas leves são aquelas que o esforço físico é normal, fazendo com que a respiração seja normal.

Domínio 1 – Atividade Física no Trabalho

Este domínio inclui as atividades que você faz no seu trabalho remunerado ou voluntário, e as atividades na universidade, faculdade ou escola (trabalho intelectual). Não incluir tarefas domésticas, cuidar do jardim e da casa ou tomar conta da sua família. Estas serão incluídas no Domínio 3.

51a - Atualmente você tem ocupação remunerada ou faz trabalho fora de sua casa?

( ) Sim ( ) Não – Caso você responda não. Vá para o Domínio 2: Transporte

As próximas questões relacionam-se com toda a atividade física que você faz em uma semana normal/habitual, como parte do seu trabalho remunerado ou voluntário. Não inclua o transporte para o trabalho. Pense apenas naquelas atividades que durem pelo menos 10 minutos contínuos dentro de seu trabalho:

51b - Quantos dias e qual o tempo (horas e minutos) durante uma semana normal você realiza atividades VIGOROSAS como: trabalho de construção pesada, levantar e transportar objetos pesados, cortar lenha, serrar madeira, cortar grama, pintar casa, cavar valas ou buracos, subir escadas como parte do seu trabalho remunerado ou voluntário, por pelo menos 10 MINUTOS CONTÍNUOS?

_____horas _____ minutos_______ dias por semana ( ) Nenhum. Vá para a questão 51c.

Dia da semana/turno		Segunda-feira	Terça-feira	Quarta-feira	Quinta-feira	Sexta-feira	Sábado	Domingo
Tempo horas/minutos	Manhã							
	Tarde							
	Noite							

continue...

...Continuation

Appendix 1. Instrumento de Avaliação de Saúde do Idoso

Domínio 1 – Atividade Física no Trabalho

51c - Quantos dias e qual o tempo (horas e minutos) durante uma semana normal você realiza atividades MODERADAS como: trabalho de construção pesada, levantar e transportar objetos pesados, cortar lenha, serrar madeira, cortar grama, pintar casa, cavar valas ou buracos, subir escadas como parte do seu trabalho remunerado ou voluntário, por pelo menos 10 MINUTOS CONTÍNUOS?

____horas ____ minutos ______ dias por semana ( ) Nenhum. Vá para a questão 51d.

Dia da semana/turno		Segunda-feira	Terça-feira	Quarta-feira	Quinta-feira	Sexta-feira	Sábado	Domingo
Tempo horas/minutos	Manhã							
	Tarde							
	Noite							

51d - Quantos dias e qual o tempo (horas e minutos) durante uma semana normal você CAMINHA, NO SEU TRABALHO remunerado ou voluntário, por pelo menos 10 MINUTOS CONTÍNUOS? Por favor, não inclua o caminhar como forma de transporte para ir ou voltar do trabalho ou do local que você é voluntário.

____horas ____ minutos ______ dias por semana ( ) Nenhum. Vá para o Domínio 2 - Transporte.

Dia da semana/turno		Segunda-feira	Terça-feira	Quarta-feira	Quinta-feira	Sexta-feira	Sábado	Domingo
Tempo horas/minutos	Manhã							
	Tarde							
	Noite							

Domínio 2 – Atividade Física Como Meio de Transporte

Estas questões se referem à forma normal como você se desloca de um lugar para outro, incluindo seu grupo de convivência para idosos, igreja, supermercado, trabalho, cinema, lojas e outros.

52a - Quantos dias e qual tempo (horas e minutos) durante uma semana normal você ANDA DE ÔNIBUS E CARRO/MOTO?

____horas ____ minutos ______ dias por semana ( ) Nenhum. Vá para a questão 52b.

Dia da semana/turno		Segunda-feira	Terça-feira	Quarta-feira	Quinta-feira	Sexta-feira	Sábado	Domingo
Tempo horas/minutos	Manhã							
	Tarde							
	Noite							

Agora pense em relação a caminhar ou pedalar para ir de um lugar a outro em uma semana normal.

52b - Quantos dias e qual o tempo (horas e minutos) durante uma semana normal você ANDA DE BICICLETA para ir de um lugar para outro por pelo menos 10 minutos contínuos? (Não inclua pedalar por lazer ou exercício).

____horas ____ minutos ______ dias por semana ( ) Nenhum. Vá para a questão 52c.

Dia da semana/turno		Segunda-feira	Terça-feira	Quarta-feira	Quinta-feira	Sexta-feira	Sábado	Domingo
Tempo horas/minutos	Manhã							
	Tarde							
	Noite							

52c - Quantos dias e qual o tempo (horas e minutos) durante uma semana normal você CAMINHA para ir de um lugar para outro, como: ir ao grupo de convivência para idosos, igreja, supermercado, médico, banco, visita a amigo, vizinho e parentes por pelo menos 10 minutos contínuos (NÃO INCLUA as caminhadas por lazer ou exercício físico).

____horas ____ minutos ______ dias por semana ( ) Nenhum. Vá para o Domínio 3.

Dia da semana/turno		Segunda-feira	Terça-feira	Quarta-feira	Quinta-feira	Sexta-feira	Sábado	Domingo
Tempo horas/minutos	Manhã							
	Tarde							
	Noite							

Domínio 3 – Atividade Física em Casa ou Apartamento: Trabalho, Tarefas Domésticas e Cuidar da Família

Esta parte inclui as atividades físicas que você faz em uma semana normal/habitual dentro e ao redor da sua casa ou apartamento. Por exemplo: trabalho doméstico, cuidar do jardim, cuidar do quintal, trabalho de manutenção da casa e para cuidar da sua família. Novamente pense somente naquelas atividades físicas com duração por pelo menos 10 minutos contínuos.

53a - Quantos dias e qual o tempo (horas e minutos) durante uma semana normal você faz atividades físicas VIGOROSAS AO REDOR DE SUA CASA OU APARTAMENTO (QUINTAL OU JARDIM) como: carpir, cortar lenha, serrar madeira, pintar casa, levantar e transportar objetos pesados, cortar grama por pelo menos 10 MINUTOS CONTÍNUOS?

_____ horas _____ minutos ____ dias por semana ( ) Nenhum. Vá para a questão 53b.

continue...

...Continuation

Appendix 1. Instrumento de Avaliação de Saúde do Idoso

Domínio 3 – Atividade Física em Casa ou Apartamento: Trabalho, Tarefas Domésticas e Cuidar da Família

Dia da semana/turno		Segunda-feira	Terça-feira	Quarta-feira	Quinta-feira	Sexta-feira	Sábado	Domingo
Tempo horas/minutos	Manhã							
	Tarde							
	Noite							

53b - Quantos dias e qual o tempo (horas e minutos) durante uma semana normal você faz atividades MODERADAS DENTRO da sua casa ou apartamento como: carregar pesos leves, limpar vidros e/ou janelas, lavar roupas a mão, limpar banheiro e o chão por pelo menos 10 minutos contínuos?

_____ horas _____ minutos ____ dias por semana ( ) Nenhum. Vá para o domínio 4.

Dia da semana/turno		Segunda-feira	Terça-feira	Quarta-feira	Quinta-feira	Sexta-feira	Sábado	Domingo
Tempo horas/minutos	Manhã							
	Tarde							
	Noite							

Domínio 4 — Atividades Físicas de Recreação, Esporte, Exercício e de Lazer

Este domínio se refere às atividades físicas que você faz em sua semana normal/habitual unicamente por recreação, esporte, exercício ou lazer. Novamente pense somente nas atividades físicas que você faz por pelo menos 10 minutos contínuos. Por favor, não inclua atividades que você já tenha citado.

54a - Sem contar qualquer caminhada que você tenha dito anteriormente, quantos dias e qual o tempo (horas/minutos) durante uma semana normal você CAMINHA (exercício físico) no seu tempo livre por PELO MENOS 10 MINUTOS CONTÍNUOS?

_____ horas _____ minutos ____ dias por semana ( ) Nenhum. Vá para o domínio 54b.

Dia da semana/turno		Segunda-feira	Terça-feira	Quarta-feira	Quinta-feira	Sexta-feira	Sábado	Domingo
Tempo horas/minutos	Manhã							
	Tarde							
	Noite							

54b - Quantos dias e qual o tempo (horas e minutos) durante uma semana normal, você faz atividades VIGOROSAS no seu tempo livre como: correr, nadar rápido, musculação, canoagem, remo, enfim esportes em geral por pelo menos 10 minutos contínuos?

_____ horas _____ minutos ____ dias por semana ( ) Nenhum. Vá para o domínio 54c.

Dia da semana/turno		Segunda-feira	Terça-feira	Quarta-feira	Quinta-feira	Sexta-feira	Sábado	Domingo
Tempo horas/minutos	Manhã							
	Tarde							
	Noite							

54c - Quantos dias e qual o tempo (horas e minutos) durante uma semana normal, você faz atividades MODERADAS no seu tempo livre como: pedalar em ritmo moderado, jogar voleibol recreativo, fazer hidroginástica, ginástica para a terceira idade, dançar por pelo menos 10 minutos contínuos?

_____ horas _____ minutos ____ dias por semana ( ) Nenhum. Vá para o domínio 5.

Dia da semana/turno		Segunda-feira	Terça-feira	Quarta-feira	Quinta-feira	Sexta-feira	Sábado	Domingo
Tempo horas/minutos	Manhã							
	Tarde							
	Noite							

Domínio 5 – Tempo Gasto Sentado

Estas últimas questões são sobre o tempo que você permanece sentado em diferentes locais como, por exemplo, em casa, no grupo de convivência para idosos, no consultório médico e outros. Isso inclui o tempo sentado, enquanto descansa, assiste televisão, faz trabalhos manuais, visita amigos e parentes, faz leituras, telefonemas e realiza as refeições. Não inclua o tempo gasto sentado durante o transporte em ônibus, carro, trem e metrô.

55 - Quanto tempo, no total, você gasta sentado durante UM DIA de semana normal?

UM DIA: _______ horas e _____ minutos.

Dia da semana	Tempo horas/minutos		
Um dia	Manhã	Tarde	Noite

continue...

...Continuation

Appendix 1. Instrumento de Avaliação de Saúde do Idoso

**Domínio 6 — Barreiras para Prática de Atividade Física**		
**Barreiras a prática de atividade física**	**Sim**	**Não**
56 - Falta de tempo?		
57 - Preguiça?		
58 - Não tinha local adequado?		
59 - Se machucou?		
60 - Falta de dinheiro?		
61 - Falta de companhia?		
62 - Achava chato/não gostava?		
63 - Se sente velho(a) demais?		
64 - Possui alguma lesão ou doença que atrapalhe na hora de fazer atividade física?		
65 - Gosta de fazer atividade física?		
66 - Sente preguiça ou cansaço para fazer atividades físicas?		
67 - Sente medo?		
OBS - Outro__________________		

Domínio 7 — Atividade Física no Lazer

As atividades físicas no lazer são esforços físicos realizados no tempo livre, nesse sentido, responda a questão abaixo:

68 - Como você classificaria sua atividade física de lazer?

(1) leve – caminhar, pedalar ou dançar mais ou igual que 3 horas por semana.

(2) moderada – correr, fazer ginástica ou praticar esportes mais ou igual que 3 horas por semana.

(3) intensa – treinamento para competição.

(4) não tem – o lazer não inclui atividade física.

Domínio 8 — Estágio de Mudança no Comportamento para a Atividade Física no Lazer

69 - O(A) Sr.(a) realiza em seu tempo livre algum tipo de atividade física moderada? (por exemplo: caminhar rápido, esportes, ginástica de academia, faxina, subir escadas ou qualquer outra atividade física de esforço similar a estas)

1 ( ) Sim 2 ( ) Não

70 - Em quantos dias de uma semana normal, em seu tempo livre, o(a) Sr.(a) realiza atividades físicas moderadas, que somadas totalizam pelo menos 30 minutos por dia?

( ) 0 ( ) 1 ( ) 2 ( ) 3 ( ) 4 ( ) 5 ( ) 6 ( ) 7

71 - Considera-se FISICAMENTE ATIVO o adulto que acumula pelo menos 30 minutos diários de atividades físicas em 5 dias ou mais da semana. Em relação a seus hábitos de práticas de atividade físicas no lazer o(a) Sr (a) considera que:

1 ( ) Sou fisicamente ativo HÁ MAIS DE 6 MESES.

2 ( ) Sou fisicamente ativo HÁ MENOS DE 6 MESES.

3 ( ) Não tenho este hábito, mas pretendo me tornar fisicamente ativo nos próximos 30 DIAS.

4 ( ) Não tenho este hábito, mas pretendo me tornar fisicamente ativo nos próximos 6 MESES.

5 ( ) Não tenho este hábito e não pretendo me tornar fisicamente ativo nos próximos 6 MESES.

Bloco 4

IV. Capacidade funcional[19,20]

Precisamos entender as dificuldades que algumas pessoas têm em realizar certas atividades que são importantes para a vida diária devido a algum problema de saúde. O(A) Sr.(a) poderia me dizer, por favor, se encontra alguma dificuldade (atualmente) em fazer cada uma das seguintes atividades que vou dizer. Não considere qualquer problema que o(a) Sr.(a) espera que dure menos de 3 meses.

O idoso é: (1) deambulante (2) acamado (3) cadeirante (8) NS

Tarefas de atividades físicas mais elaboradas	Sim	Não	Não pode	Não faz	NR
72a - Tem dificuldade em correr ou trotar 1,5km ou 15 quadras?	1	2 - Vá para a 2	3	4	9
72b - Tem dificuldade em andar várias ruas (quadras)?	1	2 - Vá para a 2	3	4	9
72c - Tem dificuldade em andar uma rua (quadra)?	1	2	3	4	9
73 - Tem dificuldade em ficar sentado(a) durante 2 horas?	1	2	3	4	9
74 - Tem dificuldade em se levantar de uma cadeira depois de ficar sentado(a) durante longo período?	1	2	3	4	9

continue...

...Continuation

Appendix 1. Instrumento de Avaliação de Saúde do Idoso

**Bloco 4**					
**IV. Capacidade funcional**[19,20]					
75 - Encontra alguma dificuldade em subir vários lances de escada sem parar para descansar?	1	2 - Vá para a 6	3	4	9
76 - Tem alguma dificuldade em subir um andar pelas escadas sem descansar?	1	2	3	4	9
77 - Tem alguma dificuldade em se curvar, se ajoelhar ou se agachar?	1	2	3	4	9
78 - Tem alguma dificuldade para estender seus braços acima dos ombros?	1	2	3	4	9
79 - Tem alguma dificuldade para puxar ou empurrar grandes objetos, como uma poltrona?	1	2	3	4	9
80 - Encontra alguma dificuldade em levantar ou carregar pesos maiores que 5kg, como uma sacola de compras pesada?	1	2	3	4	9
81 - Tem dificuldade em levantar uma moeda de uma mesa?	1	2	3	4	9

**Atividade**	**Avaliação**	
82 - O(A) Sr.(a) consegue usar o telefone?	Sem ajuda	1 ( )
	Com ajuda parcial	2 ( )
	Não consegue	3 ( )
83 - O(A) Sr.(a) consegue ir a locais distantes, usando algum transporte, sem necessidade de planejamentos especiais?	Sem ajuda	1 ( )
	Com ajuda parcial	2 ( )
	Não consegue	3 ( )
84 - O(A) Sr.(a) consegue fazer compras?	Sem ajuda	1 ( )
	Com ajuda parcial	2 ( )
	Não consegue	3 ( )
85 - O(A) Sr.(a) consegue preparar as suas próprias refeições?	Sem ajuda	1 ( )
	Com ajuda parcial	2 ( )
	Não consegue	3 ( )
86 - O(A) Sr.(a) consegue arrumar a casa?	Sem ajuda	1 ( )
	Com ajuda parcial	2 ( )
	Não consegue	3 ( )
87 - O(A) Sr.(a) consegue fazer trabalhos manuais domésticos, como pequenos reparos?	Sem ajuda	1 ( )
	Com ajuda parcial	2 ( )
	Não consegue	3 ( )
88 - O(A) Sr.(a) consegue lavar e passar sua roupa?	Sem ajuda	1 ( )
	Com ajuda parcial	2 ( )
	Não consegue	3 ( )
89 - O(A) Sr.(a) consegue tomar seus remédios na dose e horários corretos?	Sem ajuda	1 ( )
	Com ajuda parcial	2 ( )
	Não consegue	3 ( )
90 - O(A) Sr.(a) consegue cuidar de suas finanças?	Sem ajuda	1 ( )
	Com ajuda parcial	2 ( )
	Não consegue	3 ( )
Total:	_______pontos	

**Bloco 5**
**V. Estado de saúde**[21]
A.
91 - Agora gostaria de lhe fazer algumas perguntas sobre a sua saúde. O(A) Sr.(a) diria que sua saúde é excelente, muito boa, boa, regular ou má?
(1) Excelente (2) Muito boa (3) Boa (4) Regular (5) Má (8) NS (9) NR
92 - Comparando sua saúde de hoje com a de 12 meses atrás, o(a) Sr.(a) diria que agora sua saúde é melhor, igual ou pior do que estava então?
(1) Melhor (2) Igual (3) Pior (8) NS (9) NR
93 - Em comparação com outras pessoas de sua idade, o(a) Sr.(a) diria que sua saúde é melhor, igual ou pior?
(1) Melhor (2) Igual (3) Pior (8) NS (9) NR

continue...

...Continuation

Appendix 1. Instrumento de Avaliação de Saúde do Idoso

Bloco 5

V. Estado de saúde[21]

94 - Você tem algum dos problemas de saúde listados abaixo?

Diabetes	0 ( ) Sim	1 ( ) Não	Distúrbio do sono	0 ( ) Sim	1 ( ) Não
Colesterol alto	0 ( ) Sim	1 ( ) Não	Hanseníase	0 ( ) Sim	1 ( ) Não
Parkinson	0 ( ) Sim	1 ( ) Não	Tuberculose	0 ( ) Sim	1 ( ) Não
Pressão alta	0 ( ) Sim	1 ( ) Não	DAD	0 ( ) Sim	1 ( ) Não
Câncer	0 ( ) Sim	1 ( ) Não	Embolia pulmonar	0 ( ) Sim	1 ( ) Não
Cardiopatia	0 ( ) Sim	1 ( ) Não	Dores de coluna	0 ( ) Sim	1 ( ) Não
Doença da tireoide	0 ( ) Sim	1 ( ) Não	Doença de Alzheimer	0 ( ) Sim | 1 ( ) Não	
Histórico de queda	0 ( ) Sim	1 ( ) Não	Outras		

DAD: doença articular degenerativa.

B. Uso de medicamentos

Gostaria de tomar nota dos remédios e outras coisas que o(a) Sr.(a) está tomando ou usando atualmente.

95 - O(A) Sr.(a) poderia me mostrar os remédios que atualmente está usando ou tomando?

(1) Sim (2) Não (3) Não toma medicamentos - Vá para seção V B

96 - Caso a pessoa entrevistada não tenha mostrado os remédios, pergunte: "O(A) Sr.(a) poderia me dizer o nome dos remédios de uso contínuo que está usando ou tomando?"

*Anotar apenas os 5 principais e fazer observação no caso de maior número.

1. ____________________
2. ____________________
3. ____________________
4. ____________________
5. ____________________

97 - Quem o receitou?

(1) Médico (2) Farmacêutico (3) Enfermeira (4) O(A) Sr.(a) mesmo(a)
(5) Outro (8) NS (9) NR

1. ____________( )
2. ____________( )
3. ____________( )
4. ____________( )
5. ____________( )

98 - Há quanto tempo usa este medicamento de maneira contínua?

(98) NS___ (99) NR

1. ____________
2. ____________
3. ____________
4. ____________
5. ____________

99 - Como obteve ou quem pagou pelo remédio, na última vez que o comprou?

(1) Seguro social (2) Outro seguro público
(3) Seguro particular (4) Do seu próprio bolso
(5) Filhos pagam (6) Outro. Qual?_____
(8) NS (9) NR

1. ____________( )
2. ____________( )
3. ____________( )
4. ____________( )
5. ____________( )

100 - Atualmente, o(a) Sr.(a) toma (outros) remédios naturais, como ervas ou produtos homeopáticos para cuidar da sua saúde?

(1) Sim Volte para 2 e anote (2) Não (8) NS (9) NR

continue...

...Continuation

Appendix 1. Instrumento de Avaliação de Saúde do Idoso

C. Uso e acesso aos serviços

101 - Que tipo de seguro de saúde o(a) Sr.(a) tem? (Assinale todas as respostas mencionadas)

(1) Plano de Saúde Privado

(2) Seguro Público (Sistema Único de Saúde - SUS)

(3) Outro:__________________

(4) Nenhum (8) NS (9) NR

Bloco 6

VI. Saúde mental[22-27]

A.

Neste estudo, estamos investigando como o(a) Sr.(a) se sente a respeito de alguns problemas de saúde.

Gostaríamos de começar com algumas perguntas sobre sua memória.

102 - Como o(a) Sr.(a) avalia sua memória atualmente? (leia as opções e responda)

(1) Excelente (2) Muito boa (3) Boa (4) Regular (5) Má (8) NS (9) NR

103 - Comparando com 1 ano atrás, o(a) Sr.(a) diria que agora sua memória é melhor, igual ou pior?

(1) Melhor (2) Igual (3) Pior (8) NS (9) NR

104 - Por favor, me diga a data de hoje (Pergunte mês, dia, ano e dia da semana. Anote um ponto em cada resposta correta).

Códigos:		Correto
Segunda feira 01	Mês |__|__|	( )
Terça feira 02	Dia do mês |__|__|	( )
Quarta feira 03	Ano |__|__|__|__|	( )
Quinta feira 04	Dia da semana |__|__|	( )
Sexta feira 05		Total ( )
Sábado 06		
Domingo 07		

105 - Agora vou lhe dar o nome de três objetos. Quando eu terminar lhe pedirei que repita em voz alta todas as palavras que puder lembrar, em qualquer ordem. Guarde quais são porque vou voltar a perguntar mais adiante. O(A) Sr.(a) tem alguma pergunta?

(Leia os nomes dos objetos devagar e de forma clara somente uma vez e anote).

Se o entrevistado não acertar as três palavras:

1) Repita todos os objetos até que o entrevistado os aprenda, máximo de repetições: 5 vezes.

2) Anote o número de repetições que teve que fazer.

3) Nunca corrija a primeira parte.

4) Anota-se um ponto por cada objeto lembrado e zero para os não lembrados.

ÁRVORE ( )	(1) Lembrou
MESA ( )	(0) Não lembrou
CACHORRO ( )	NÚMERO DE REPETIÇÕES: _______

Total: ( )

106 - "Agora peço-lhe que me diga quantos são 30 menos 3 e depois ao número encontrado volta a tirar 3 e repete assim até eu lhe dizer para parar". (1 ponto por cada resposta correta. Se der uma errada, mas depois continuar a subtrair bem, consideram-se as seguintes como corretas. Parar ao fim de 5 respostas)

27_ 24_ 21 _ 18_ 15_

Total: ( )

107 - Vou lhe dar um papel e quando eu o entregar, apanhe o papel com sua mão direita, dobre-o na metade com as duas mãos e coloque-o sobre suas pernas (Passe o papel e anote 1 ponto para cada ação correta).

Pega o papel com a mão direita ( )	Ação correta: 1 ponto
Dobra na metade com as duas mãos ( )	Ação incorreta: 0
Coloca o papel sobre as pernas ( )	

Total: ( )

continue...

...Continuation

Appendix 1. Instrumento de Avaliação de Saúde do Idoso

Bloco 6

VI. Saúde mental[22-27]

A.

108 - Há alguns minutos li uma série de três palavras e o(a) Sr.(a) repetiu as palavras que lembrou. "Veja se consegue dizer as três palavras que pedi há pouco para decorar".

(1 ponto por cada resposta correta).

ÁRVORE ( ) Lembrou: 1

MESA ( ) Não lembrou: 0

CACHORRO ( )

Total: ( )

109 - Por favor, copie este desenho. Entregue ao entrevistado o desenho com os círculos que se cruzam. A ação está correta se os círculos não se cruzarem mais do que a metade. Anote um ponto se o desenho estiver correto.

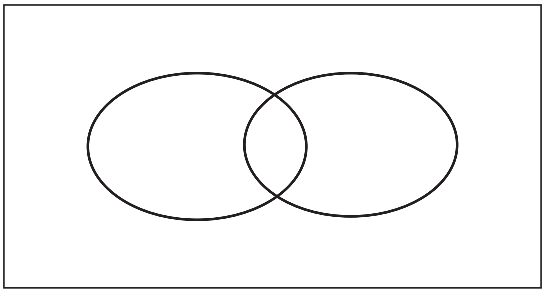

Correto: ( ) Total: ( )

110 - NÃO LER! FILTRO- Some as respostas corretas anotadas nas perguntas 41 a 46 e anote o total (a pontuação máxima é 19)

(1) a soma é 13 ou mais.

(2) a soma é 12 ou menos.

111 - Alguma outra pessoa que mora nesta casa poderia ajudar-nos a responder algumas perguntas?

(1) SIM (anote o nome do informante e aplique a escala abaixo).

(2) NÃO (avalie com o supervisor se a entrevista pode continuar só com a pessoa entrevistada).

Mostre ao informante a seguinte cartela com as opções e leia as perguntas. Anote a pontuação como segue:

(0) Sim, é capaz.

(0) Nunca o fez, mas poderia fazer agora.

(1) Com alguma dificuldade, mas faz.

(1) Nunca fez e teria dificuldade agora.

(2) Necessita de ajuda.

(3) Não é capaz.

112a - (NOME) é capaz de cuidar do seu próprio dinheiro? ( )

112b - (NOME) é capaz de fazer compras sozinho (por exemplo de comida e roupa)? ( )

112c - (NOME) é capaz de esquentar água para café ou chá e apagar o fogo? ( )

112d - (NOME) é capaz de preparar comida? ( )

112e - (NOME) é capaz de manter-se a par dos acontecimentos e do que se passa na vizinhança? ( )

112f - (NOME) é capaz de prestar atenção, entender e discutir um programa de rádio, televisão ou um artigo do jornal? ( )

112g - (NOME) é capaz de lembrar de compromissos e acontecimentos familiares? ( )

112h - (NOME) é capaz de cuidar de seus próprios medicamentos? ( )

113 - Some os pontos das perguntas de 48a ao 48h e anote no "TOTAL". Total: ( )

(1) A soma é 6 ou mais (continue a entrevista com ajuda do informante substituto e revise a Seção.

(2) A soma é 5 ou menos (continue a entrevista com o entrevistado. Caso a pessoa necessite de ajuda para responder algumas perguntas, continue com um informante auxiliar).

continue...

...Continuation

Appendix 1. Instrumento de Avaliação de Saúde do Idoso

**B. Saúde Mental – Questionário *Brasil Old Age Schedule* (BOAS)**		
É bastante comum as pessoas terem problemas de memória quando começam a envelhecer. Deste modo, eu gostaria de lhe fazer algumas perguntas sobre este assunto. Algumas perguntas talvez não sejam apropriadas para o(a) Sr.(a), outras bastantes inadequadas. No entanto, eu gostaria que o(a) Sr.(a) levasse em conta que tenho que fazer as mesmas perguntas para todas as pessoas.		
114 - Esta pesquisa está sendo realizada pela Universidade Estadual do Sudoeste da Bahia (UESB). Eu gostaria que o(a) Sr.(a) repetisse para mim este nome e guardasse na memória.		
1. Repete UESB ou algo próximo.		
2. Não consegue/não repete/não responde.		
115 - Em que ano o(a) Sr.(a) nasceu? Ano do nascimento:_________________		
1. Ano do nascimento aparentemente correto.		
2. Informa ano do nascimento que não corresponde à impressão do observador; ou é inconsistente com a data anteriormente obtida; ou é obviamente errada; ou não sabe ou fornece resposta incompleta.		
116 - Qual é o endereço de sua casa?		
1. Informa endereço correto.		
2. Informa endereço incorreto ou não sabe ou fornece informação incompleta.		
117 - Há quanto tempo o(a) Sr.(a) mora neste endereço?_________________ anos.		
1. Informação sobre tempo de residência aproximadamente correto/plausível.		
2. Informa tempo de residência obviamente errado ou não sabe.		
118 - O(a) Sr.(a) sabe o nome do atual presidente do Brasil? Registre:_________________		
1. Nome do presidente correto/quase correto.		
2. Informa nome incorreto ou não recorda nome do presidente.		
119 - Em que mês do ano nós estamos? Mês do ano:_________________		
1. Mês correto.		
2. Informa incorretamente o mês ou não sabe.		
120 - Em que ano nós estamos? Registre:_________________		
1. Ano correto.		
2. Informa incorretamente o ano ou não sabe.		
**C. *Self-Report Questionnaire* (SRQ-20)**		
As próximas questões estão relacionadas a situações que você pode ter vivido nos últimos 30 DIAS. Se você acha que a questão se aplica a você e você sentiu a situação descrita nos últimos 30 DIAS responda SIM. Por outro lado, se a questão não se aplica a você e você não sentiu a situação, responda NÃO. Se você está incerto sobre como responder uma questão, por favor, dê a melhor resposta que você puder.		
	Não	Sim
121 - Tem dores de cabeça frequentemente?		
122 - Tem falta de apetite?		
123 - Dorme mal?		
124 - Assusta-se com facilidade?		
125 - Tem tremores nas mãos?		
126 - Sente-se nervoso(a), tenso(a) ou preocupado(a)?		
127 - Tem má digestão?		
128 - Tem dificuldade de pensar com clareza?		
129 - Tem se sentido triste ultimamente?		
130 - Tem chorado mais do que de costume?		
131 - Encontra dificuldade de realizar, com satisfação, suas tarefas diárias?		
132 - Tem dificuldade para tomar decisões?		
133 - Seu trabalho diário lhe causa sofrimento?		
134 - É incapaz de desempenhar um papel útil em sua vida?		
135 - Tem perdido o interesse pelas coisas?		
136 - Você se sente pessoa inútil em sua vida?		
137 - Tem tido ideia de acabar com a vida?		
138 - Sente-se cansado(a) o tempo todo?		
139 - Tem sensações desagradáveis no estômago?		
140 - Você se cansa com facilidade?		

0: não; 1: sim.

continue...

...Continuation

Appendix 1. Instrumento de Avaliação de Saúde do Idoso

**D. Escala de Depressão Geriátrica de Yesavage – Versão Reduzida (GDS-15)**		
	Sim	Não
141 - Você está satisfeito com a sua vida?		
142 - Você deixou de lado muitas de suas atividades e interesses?		
143 - Você sente que sua vida está vazia?		
144 - Você sente-se aborrecido com frequência?		
145 - Está você de bom humor na maioria das vezes?		
146 - Você teme que algo de ruim lhe aconteça?		
147 - Você se sente feliz na maioria das vezes?		
148 - Você se sente frequentemente desamparado?		
149 - Você prefere permanecer em casa do que sair e fazer coisas novas?		
150 - Você sente que tem mais problemas de memória que antes?		
151 - Você pensa que é maravilhoso estar vivo?		
152 - Você se sente inútil?		
153 - Você se sente cheio de energia?		
154 - Você sente que sua situação é sem esperança?		
155 - Você pensa de que a maioria das pessoas estão melhores do que você?		

Bloco 7[21]

***VII. World Health Organization Quality of Life Assessment for Older Adults* (WHOQOL-OLD)**

Instruções:

Este questionário pergunta a respeito dos seus pensamentos, sentimentos e sobre certos aspectos de sua qualidade de vida, e aborda questões que podem ser importantes para você como membro mais velho da sociedade.

Por favor, responda todas as perguntas. Se você não está seguro a respeito de que resposta dar a uma pergunta, por favor, escolha a que lhe parece mais apropriada. Esta pode ser muitas vezes a sua primeira resposta.

Por favor, tenha em mente os seus valores, esperanças, prazeres e preocupações. Pedimos que pense na sua vida nas 2 últimas semanas.

Por exemplo, pensando nas 2 últimas semanas, uma pergunta poderia ser:

O quanto você se preocupa com o que o futuro poderá trazer?

Nada	Muito pouco	Mais ou menos	Bastante	Extremamente
1	2	3	4	5

Você deve circular o número que melhor reflete o quanto você se preocupou com o seu futuro durante as 2 últimas semanas. Então você circularia o número 4 se você se preocupou com o futuro "Bastante", ou circularia o número 1 se não tivesse se preocupado "Nada" com o futuro. Por favor, leia cada questão, pense no que sente e circule o número na escala que seja a melhor resposta para você para cada questão.

Muito obrigado(a) pela sua colaboração!

As seguintes questões perguntam sobre o quanto você tem tido certos sentimentos nas últimas 2 semanas.

156 - Até que ponto as perdas nos seus sentidos (por exemplo, audição, visão, paladar, olfato e tato), afetam a sua vida diária?

Nada	Muito pouco	Mais ou menos	Bastante	Extremamente
1	2	3	4	5

157 - Até que ponto a perda de, por exemplo, audição, visão, paladar, olfato, tato, afeta a sua capacidade de participar em atividades?

Nada	Muito pouco	Mais ou menos	Bastante	Extremamente
1	2	3	4	5

158 - Quanta liberdade você tem de tomar as suas próprias decisões?

Nada	Muito pouco	Mais ou menos	Bastante	Extremamente
1	2	3	4	5

159 - Até que ponto você sente que controla o seu futuro?

Nada	Muito pouco	Mais ou menos	Bastante	Extremamente
1	2	3	4	5

160 - O quanto você sente que as pessoas ao seu redor respeitam a sua liberdade?

Nada	Muito pouco	Mais ou menos	Bastante	Extremamente
1	2	3	4	5

161 - Quão preocupado você está com a maneira pela qual irá morrer?

Nada	Muito pouco	Mais ou menos	Bastante	Extremamente
1	2	3	4	5

continue...

...Continuation

Appendix 1. Instrumento de Avaliação de Saúde do Idoso

Bloco 7[21]<br>***VII. World Health Organization Quality of Life Assessment for Older Adults* (WHOQOL-OLD)**				
162 - O quanto você tem medo de não poder controlar a sua morte?				
Nada	Muito pouco	Mais ou menos	Bastante	Extremamente
1	2	3	4	5
163 - O quanto você tem medo de morrer?				
Nada	Muito pouco	Mais ou menos	Bastante	Extremamente
1	2	3	4	5
164 - O quanto você teme sofrer dor antes de morrer?				
Nada	Muito pouco	Mais ou menos	Bastante	Extremamente
1	2	3	4	5
As seguintes questões perguntam sobre quão completamente você fez ou se sentiu apto a fazer algumas coisas nas 2 últimas semanas.				
165 - Até que ponto o funcionamento dos seus sentidos (por exemplo, audição, visão, paladar, olfato e tato) afeta a sua capacidade de interagir com outras pessoas?				
Nada	Muito pouco	Mais ou menos	Bastante	Extremamente
1	2	3	4	5
166 - Até que ponto você consegue fazer as coisas que gostaria de fazer?				
Nada	Muito pouco	Mais ou menos	Bastante	Extremamente
1	2	3	4	5
167 - Até que ponto você está satisfeito com as suas oportunidades para continuar alcançando outras realizações na sua vida?				
Nada	Muito pouco	Mais ou menos	Bastante	Extremamente
1	2	3	4	5
168 - O quanto você sente que recebeu o reconhecimento que merece na sua vida?				
Nada	Muito pouco	Mais ou menos	Bastante	Extremamente
1	2	3	4	5
169 - Até que ponto você sente que tem o suficiente para fazer em cada dia?				
Nada	Muito pouco	Mais ou menos	Bastante	Extremamente
1	2	3	4	5
As seguintes questões pedem a você que diga o quanto você se sentiu satisfeito, feliz ou bem sobre vários aspectos de sua vida nas 2 últimas semanas.				
170 - Quão satisfeito você está com aquilo que alcançou na sua vida?				
Muito insatisfeito	Insatisfeito	Nem satisfeito nem insatisfeito	Satisfeito	Muito satisfeito
1	2	3	4	5
171 - Quão satisfeito você está com a maneira com a qual você usa o seu tempo?				
Muito insatisfeito	Insatisfeito	Nem satisfeito nem insatisfeito	Satisfeito	Muito satisfeito
1	2	3	4	5
172 - Quão satisfeito você está com o seu nível de atividade?				
Muito insatisfeito	Insatisfeito	Nem satisfeito nem insatisfeito	Satisfeito	Muito satisfeito
1	2	3	4	5
173 - Quão satisfeito você está com as oportunidades que tem para participar de atividades da comunidade?				
Muito insatisfeito	Insatisfeito	Nem satisfeito nem insatisfeito	Satisfeito	Muito satisfeito
1	2	3	4	5
174 - Quão feliz você está com as coisas que você pode esperar daqui para frente?				
Muito insatisfeito	Insatisfeito	Nem satisfeito nem insatisfeito	Satisfeito	Muito satisfeito
1	2	3	4	5
175 - Como você avaliaria o funcionamento dos seus sentidos (por exemplo, audição, visão, paladar, olfato e tato)?				
Muito insatisfeito	Insatisfeito	Nem satisfeito nem insatisfeito	Satisfeito	Muito satisfeito
1	2	3	4	5
As seguintes questões se referem a qualquer relacionamento íntimo que você possa ter. Por favor, considere estas questões em relação a um companheiro ou uma pessoa próxima com a qual você pode compartilhar (dividir) sua intimidade mais do que com qualquer outra pessoa em sua vida.				
176 - Até que ponto você tem um sentimento de companheirismo em sua vida?				
Nada	Muito pouco	Mais ou menos	Bastante	Extremamente
1	2	3	4	5

continue...

...Continuation

Appendix 1. Instrumento de Avaliação de Saúde do Idoso

Bloco 7[21]

***VII. World Health Organization Quality of Life Assessment for Older Adults* (WHOQOL-OLD)**

177 - Até que ponto você sente amor em sua vida?

Nada	Muito pouco	Mais ou menos	Bastante	Extremamente
1	2	3	4	5

178 - Até que ponto você tem oportunidades para amar?

Nada	Muito pouco	Mais ou menos	Bastante	Extremamente
1	2	3	4	5

179 - Até que ponto você tem oportunidades para ser amado?

Nada	Muito pouco	Mais ou menos	Bastante	Extremamente
1	2	3	4	5

Você tem algum comentário sobre o questionário?

Obrigado(a) pela sua colaboração!

Instruções:

Este questionário é sobre como você se sente a respeito de sua qualidade de vida, saúde e outras áreas de sua vida. Por favor, responda a todas as questões. Se você não tem certeza sobre que resposta dar em uma questão, por favor, escolha entre as alternativas a que lhe parece mais apropriada. Esta, muitas vezes, poderá ser sua primeira escolha. Por favor, tenha em mente seus valores, aspirações, prazeres e preocupações. Nós estamos perguntando o que você acha de sua vida, tomando como referência as 2 últimas semanas. Por exemplo, pensando nas últimas 2 semanas, uma questão poderia ser:

	Nada	Muito pouco	Médio	Muito	Completamente
Você recebe dos outros o apoio de que necessita?					

Você deve circular o número que melhor corresponde ao quanto você recebeu dos outros o apoio de que necessita nestas últimas 2 semanas. Portanto, você deve circular o número 4 se você recebeu "muito" apoio. Você deve circular o número 1 se você não recebeu "nada" de apoio.

	Nada	Muito pouco	Médio	Muito	Completamente
Você recebe dos outros o apoio de que necessita					

Por favor, leia cada questão, veja o que você acha e circule no número que lhe parece a melhor resposta.

		Muito ruim	Ruim	Nem ruim nem boa	Boa	Muito boa
180 -	Como você avaliaria sua qualidade de vida?					
		Muito insatisfeito	Insatisfeito	Nem satisfeito nem insatisfeito	Satisfeito	Muito satisfeito
181 -	Como você avaliaria sua qualidade de vida?					

As questões seguintes são sobre o quanto você tem sentido algumas coisas nas últimas 2 semanas.

		Nada	Muito pouco	Mais ou menos	Bastante	Extremamente
182 -	Em que medida você acha que sua dor (física) impede você de fazer o que você precisa?					
183 -	O quanto você precisa de algum tratamento médico para levar sua vida diária?					
184 -	O quanto você aproveita a vida?					
185 -	Em que medida você acha que a sua vida tem sentido?					
186 -	O quanto você consegue se concentrar?					
187 -	Quão seguro(a) se sente em sua vida diária?					
188 -	Quão saudável é o seu ambiente físico (clima, barulho, poluição, atrativos)?					

As questões seguintes perguntam sobre quão completamente você tem sentido ou é capaz de fazer certas coisas nestas últimas 2 semanas.

		Nada	Muito pouco	Médio	Muito	Completamente
189 -	Você tem energia suficiente para seu dia a dia?					
190 -	Você é capaz de aceitar sua aparência física?					
191 -	Você tem dinheiro suficiente para satisfazer suas necessidades?					
192 -	Quão disponíveis para você estão as informações que precisa no seu dia a dia?					
193 -	Em que medida você tem acesso a atividades de lazer?					

As questões seguintes perguntam sobre quão bem ou satisfeito você se sentiu a respeito de vários aspectos de sua vida nas últimas 2 semanas.

		Muito ruim	Ruim	Nem ruim	Bom	Muito bom
194 -	Quão bem você é capaz de se locomover?					

continue...

...Continuation

Appendix 1. Instrumento de Avaliação de Saúde do Idoso

Bloco 7[21] *VII. World Health Organization Quality of Life Assessment for Older Adults* (WHOQOL-OLD)						
		Muito insatisfeito	Insatisfeito	Nem satisfeito nem insatisfeito	Satisfeito	Muito satisfeito
195 -	Quão satisfeito(a) você está com o seu sono?					
196 -	Quão satisfeito(a) você está com sua capacidade de desempenhar as atividades do seu dia a dia?					
197 -	Quão satisfeito(a) você está com sua capacidade para o trabalho?					
198 -	Quão satisfeito(a) você está consigo mesmo?					
199 -	Quão satisfeito(a) você está com suas relações pessoais (amigos, parentes, conhecidos, colegas)?					
200 -	Quão satisfeito(a) você está com sua vida sexual?					
201 -	Quão satisfeito(a) você está com o apoio que você recebe de seus amigos?					
202 -	Quão satisfeito(a) você está com as condições do local onde mora?					
203 -	Quão satisfeito(a) você está com o seu acesso aos serviços de saúde?					
204 -	Quão satisfeito(a) você está com o seu meio de transporte?					
As questões seguintes referem-se a com que frequência você sentiu ou experimentou certas coisas nas últimas 2 semanas.						
		Nunca		Algumas vezes	Frequentemente	
205 -	Com que frequência você tem sentimentos negativos tais como mau humor, desespero, ansiedade, depressão?	1		2	3	
		Muito frequente		Sempre		
		4		5		
206 -	Alguém lhe ajudou a preencher este questionário? ______					
207 -	Quanto tempo você levou para preencher este questionário?______					

NS: não sabe; NR: não respondeu.
